# The Care of the Deciduous Teeth

**Published:** 1891-10

**Authors:** J. E. Hinkins

**Affiliations:** CHICAGO.


ARTICLE V.

### THE CARE OF THE DECIDUOUS TEETH.

BY J. E. HINKINS, CHICAGO.

Read before the Chicago Dental Society, 1891.

Man is born to troubles, and the first and not the last of them, from the infantile point of view, is the production of the first set of teeth. From soon after birth until the last of the deciduous teeth has been shed, the young heir of the universe has his mouthful of troubles. This process, lasting as a rule from the sixth to the twenty-fourth month, is accompanied by much local irritation and constitutional disturbance; objections which are not in the least mitigated, in the view of the principal sufferer, by the consideration that the process is absolutely "natural" and "physiological."

The variety of complicated diseases which may be expected about this time is equalled only by the various methods of treatment proposed, further aggravated in most cases by the soft-hearted and soft-headed mother, who loves her offspring not wisely but too well. However, between two evils even maternal anxiety must choose the less, and as 'twixt the dentist and inflamed gums she feels herself between the devil and the deep sea; chooses the former, figuratively speaking, *i. e.*, she brings her howling, kicking kid to the office of Dr. Hammerantongs for the first application to the teeth or gums. Happy is the doctor if he can overcome the hereditary and acquired prejudices of both mother and child sufficiently to enable him to take the necessary measures.

I. And first comes the dreadful question, to cut or not to cut? You all know too well to need any description the effect on female sensibilities produced by the sight of the lancet gleaming in the dentist's hand. This, however, is my rule and practice. If a child is brought to my office with a few teeth in sight, and the gums congested and very tense over the others, I sometimes lance. But it is better practice when the child is of hæmorrhagic diathesis, to lay the lancet down and take up constitutional treatment; as a mild cathartic. If the parents are homœopaths, Magnesia Phos, Creasotum, Calcarea Carb. and Phos., Chamomile, Mercurius, etc. But in giving calomel I make it an invariable rule to stop short of turning the child inside out, as I prefer to keep on the right side of the patient, that is to say, the *outside*! If, however, none of these remedies give relief, I let the family physician try his hand.

II. Decay of the deciduous teeth should be met promptly. Too many people think that they are not worth any trouble or expense since they are only temporary; but the fact is that their proper treatment is of vital importance, for here lies the foundation of the permanent teeth.

For small cavities I prefer an amalgam, since as a rule it lasts much longer and preserves the tooth better than any other fillings which I have used. If the cavity is large enough to produce any pain, I prefer either cement or gutta-percha. Any inflammation is the first reduced, of course, and sometimes the pulp must be destroyed before filling. The process of devitalizing the pulp calls for our best judgment and care, for we have to consider not only the death of the pulp but also the life of the permanent teeth so soon to follow in the same place. So closely allied are the two generations of teeth and so vascular are the tissues, that it is a prime mistake to attempt to consider or trust them independently.

When I find the pulp exposed and aching, my first application is carbolic acid, 95 per cent., on cotton, combined with morphine and cocaine which I mix together. After one or two applications of this kind I frequently find the pulp dead or mummified.

But sometimes these applications are not sufficient, and I apply arsenic, preferably the crude $As_2 O_3$. This application I always make in the morning and remove in the afternoon, on account of the diffusibility of the drug, covering the cavity with some quick setting oxy-phosphate of zinc.

In order to emphasize the importance of great care in using this powerful drug, I will here read a short extract from an article by Dr. J. J. Putnam in the *Journal of the American Medical Association*, entitled, "Arsenic as a Domestic Poison."

. "That the effect of arsenic—like those of lead and phosphorus—are in a measure dependent upon the action of the stored-up poison, is shown by the fact that the symptoms of medicinal poisoning often appear only when a given dose has been taken for some time, after which the patient is apt to show an increased sensitiveness (though this is not regularly the case.)

"A few years ago I collected a number of the severer cases of medicinal poisoning, and I give here a few of them in brief outline. The cases of paralysis are especially noteworthy because they are now known to occur frequently in arsenical poisoning of a certain grade, and, as we shall see, they reappear among the cases of 'domestic' poisoning.

"1. Gaillard: Typical arsenical paralysis following full, doses of Fowler's solution, increased to the limits of tolerance and administered for five weeks.

*Canada Med. and Surg. Journal*, 1886-87, Vol. XV., p. 716: Arsenical paralysis, ending fatally, after large doses (Mxx to xxx) of Fowler's solution. The autopsy showed the presence of neuritis.

"3. Hastings: Arsenical paralysis with neuritic symptoms, following M iij to v of Fowler's solution continued for some weeks.

"4. Gibb. Long course of arsenical treatment ending in neuritis, causing disorder and sensibility, pain and paralysis. The patient died six months later, having taken no arsenic in the interval, and traces of arsenic were found in the liver and bones.

"5. *Dublin Quarterly Journal*, Vol. XXXVI, p. 474. Miij of Fowler's solution were taken daily for ten or twelve months, at the end of which time 'symptoms of acute arsenical poisoning' came on, ending rapidly in death.

"6. C. L. Dana: Arsenical paresis with ataxia, Mxxx of Fowler's solution three times daily, in spite of the fact that the dose had been gradually increased.

"7. Hooper: M v Fowler's solution were given three times daily for eight months. Towards the end of this time the following symptoms came on and increased, ending three months later in death; conjunctivitis were œdema, tachycardia, tremor, excessive and progressive prostration, insomnia, irritation of the trachea and larynx.

"8. Jones: M v to xv of liquor arsenicals given three times daily; at the end of a month, intense gastro-intestinal irritation, scanty urine, tropical changes in the legs, sensory and motor paresis.

"9. Burne, cited by Taylor: Gr. one-twentieth of arsenious acid daily for four days; then inflammation of the stomach, delirium, debility and exhaustion.

"10. Taylor: Gr. one-thirtieth of arsenious acid taken twice daily for seven days, then, 'sickness,' irritation of the skin, and eczema over the whole body.

"11. Taylor: M x of liquor arsenicalis chloridi (said to be a very poisonous preparatiou) taken three times in the course of twenty-four hours; then, constriction in the throat, pain and irritation of the stomach and bowels, tingling and numbness of the hands and feet, with paresis, extreme depression; gradual recovery.

"12. Personal observations: M iv to v of Fowler's solution taken three times daily for six weeks, then there came on extreme prostration, pain and severe character in the extremities, widespread muscular atrophy, and paralysis, so severe that the patient was helpless for many months."

A case of arsenical poisoning which came under my personal observation is also to the point. Last December Mrs. J. called at my office to have her mouth examined. I found full upper denture and sloughing on the right side of the mouth from the median line to a little back of the canine process, and this had penetrated internally to the outer plate of the superior maxillary bone. She complained of headache, dryness of the throat and considerable stomach trouble and a conjunctivitis which was quite apparent. On close inquiry I found that she had been doing fancy needlework, and she had admitted taking the thread continually in her mouth, I suspected arsenical poisoning. Removing the diseased tissue as best I could, I bathed the parts with hydrated iron, and gave it internally in alternation with quinine. Two weeks of this treatment, with plenty of fresh air and no needlework, wrought great improvement. My suspicions were verified by a chemical analysis which showed a marked arsenical reaction from three of the highly colored threads.

III. Teeth with abscesses should be opened up to relieve the pressure, washing of the $H_2 O_2$ and dressing with some of the essential oils. Sometimes it is necessary to fill root canals with fiber of cotton dipped in oxy-chloride of zinc or carbolic acid, to be followed promptly by gutta-percha filling.

IV. The last act of the tragedy of dentistry is extraction justly dreaded by both operator and operatee. Though the hurt he does is done in kindness, yet it is scarcely to be wondered that to childhood's eyes the dentist should seem a fiend incarnate. Yet much of this is due to lack of good judgment on the part of the laity, and even sometimes we must admit on the part of the members of the dental profession. Never deceive a child. The discovery that you are capable of even so slight a falsehood as saying, "It won't hurt you at all," when you know it will, is a shock to his innocent confidence which he never forgets and never forgives. Honesty is the best policy always. There are two conditions of mind in which the child may be. He may have been told unwisely by his attendant that "It won't hurt any," or he may have been frightened nearly into hysterics by playmates who have a truly grown-up fiendishness for stirring up trouble. In either case the proper course to be pursued is a *kind*, *gentle truthfulness* which shall secure for you the child's respect and confidence. In this refusual to place confidence and liking where respect is lacking, childish ignorance teaches a much needed lesson to adult wisdom.

Just when the temporary teeth should be extracted is one of those important questions for which, unfortunately, no hard and fast rule can be laid down. Haste or carelessness in extraction are liable to produce irregularities of the subsequent teeth, which will be either permanent or remedied only at the cost of months of labor, suffering and expense. As in most of the important affairs of life, our chief guidance rests in experience and uncommon common sense, paying due heed to the condition and position of the adjoining teeth and to the general condition of the patient. For myself I follow the rule of never removing a cuspid tooth to make room for the permanent lateral incisor. I sincerely believe that this mistake has been the cause of more irregularities of the teeth than any other one thing.

Should a child be given any anæsthetic in dentistry? Not if it can be avoided, particularly if the child is in good physical condition. Much better, if possible, in all operations on the teeth to tell them kindly that it will hurt a little but only for an instant, that you will be as gentle as possible, and try to inspire the little trembling mite to courage. Many children will respond grandly to such a moral tonic, and when you have the satisfaction not only of winning your case, but far better, of having contributed a great block of granite to the building of the future man's or woman's character.

But there are many cases, particularly among the hyper-nervous children of the city, in which this course is both impractical and unadvisable. Even if you can screw the courage of a very sensible child up to the sticking point, it is better to avoid the nervous shock of extraction by a few whiffs of chloroform or gas, just enough for its primary effects.

In conclusion I will say that if we are gentle and truthful with these little ones and do the very best we can under the circumstances to save the tooth until the proper time for its removal, we have discharged our professional service faithfully.—*Dental Review.*

